# The Energy Status of Astrocytes Is the Achilles’ Heel of eIF2B-Leukodystrophy

**DOI:** 10.3390/cells10081858

**Published:** 2021-07-22

**Authors:** Melisa Herrero, Maron Daw, Andrea Atzmon, Orna Elroy-Stein

**Affiliations:** 1 Shmunis School of Biomedicine and Cancer Research, George S. Wise Faculty of Life Sciences, Tel Aviv University, Tel Aviv 69978, Israel; mherrerobocco@gmail.com (M.H.); marondaw@mail.tau.ac.il (M.D.); andreaat@tauex.tau.ac.il (A.A.); 2 Sagol School of Neuroscience, Tel Aviv University, Tel Aviv 69978, Israel

**Keywords:** astrocytes, eIF2B-leukodystrophy, translation regulation, impaired mitochondria function, energy stress, oxidative stress, ROS, AMPK, mTOR

## Abstract

Translation initiation factor 2B (eIF2B) is a master regulator of global protein synthesis in all cell types. The mild genetic Eif2b5(R132H) mutation causes a slight reduction in eIF2B enzymatic activity which leads to abnormal composition of mitochondrial electron transfer chain complexes and impaired oxidative phosphorylation. Previous work using primary fibroblasts isolated from Eif2b5^(R132H/R132H)^ mice revealed that owing to increased mitochondrial biogenesis they exhibit normal cellular ATP level. In contrast to fibroblasts, here we show that primary astrocytes isolated from Eif2b5^(R132H/R132H)^ mice are unable to compensate for their metabolic impairment and exhibit chronic state of low ATP level regardless of extensive adaptation efforts. Mutant astrocytes are hypersensitive to oxidative stress and to further energy stress. Moreover, they show migration deficit upon exposure to glucose starvation. The mutation in Eif2b5 prompts reactive oxygen species (ROS)-mediated inferior ability to stimulate the AMP-activated protein kinase (AMPK) axis, due to a requirement to increase the mammalian target of rapamycin complex-1 (mTORC1) signalling in order to enable oxidative glycolysis and generation of specific subclass of ROS-regulating proteins, similar to cancer cells. The data disclose the robust impact of eIF2B on metabolic and redox homeostasis programs in astrocytes and point at their hyper-sensitivity to mutated eIF2B. Thereby, it illuminates the central involvement of astrocytes in Vanishing White Matter Disease (VWMD), a genetic neurodegenerative leukodystrophy caused by homozygous hypomorphic mutations in genes encoding any of the 5 subunits of eIF2B.

## 1. Introduction

Translation initiation factor 2B (eIF2B), a master regulator of protein synthesis under normal and stress conditions, is a decameric complex composed of two homo-pentamers of eIF2B1-5 subunits which are evolutionary conserved from yeast to mammals [1,2]. While knock-out of *EIF2B* is lethal, hypo-active eIF2B due to hypo-morphic mutations specifically affect the phenotype of brain glial cells, although eIF2B is essential for every cell type. The most compelling evidence for the latter statement is the neurodegenerative pathology termed Vanishing White Matter Disease (VWMD) or eIF2B-leukodystrophy, caused by two mutated alleles of any of the 5 genes encoding eIF2B subunits (OMIM 306896) [3]. More than 160 different mutations associated with variable effects on eIF2B stability/enzymatic activity, lead to progressive deterioration of brain white matter, neurological symptoms and early death [4,5]. eIF2B serves as the guanine exchange factor of the translation initiation factor eIF2. At each round of translation initiation step, active GTP-bound eIF2 binds to methionine-charged initiator tRNA_i_^Met^ followed by association of the eIF2-GTP-Met-tRNA_i_^Met^ complex with 40S ribosomal subunit. Upon successful AUG recognition, eIF2-GDP is released and recycled back to eIF2-GTP by eIF2B [6,7]. The enzymatic activity of eIF2B is tightly regulated by multiple signals in response to physiological cues within the cell. For example, eIF2B is inhibited via phosphorylation by glycogen synthase kinase 3 (GSK3) [8]. Another important inhibitory mechanism under various stress conditions is mediated by phosphorylation of the alpha subunit of eIF2 by one of four different kinases (heme-regulated inhibitor, HRI; general control non-derepressible-2, GCN2; protein kinase R, PKR; and PKR-like endoplasmic reticulum kinase, PERK) which turns it into a competitive inhibitor of eIF2B. The latter marks the beginning of an expression program termed integrated stress response (ISR) which also includes feedback dephosphorylation of eIF2α [9].

For the study of VWMD, we previously generated a mouse strain homozygous for R132H mutation in Eif2b5, the gene encoding the catalytic subunit [10]. Our previous studies using wild-type (WT) and Eif2b5^R132H/R132H^ (Mut) mice include generation of transcriptome and proteome datasets from embryo fibroblasts and whole brains. These studies indicated that ~20% decrease in brain eIF2B enzymatic activity is responsible for a mixture of up- and down-regulation of numerous genes at the mRNA and/or protein levels [11,12,13]. Obviously, the omics datasets represent the anomalous homeostasis state of the mutants. Using unbiased omics-related experiments, we revealed the connection between hypo-active eIF2B and mitochondrial impairment. Specifically, an abnormal composition of electron transfer chain (ETC) complexes was discovered [12], followed by consequent biochemical analyses showing impaired oxidative phosphorylation (OXPHOS) and ATP production in primary cultures of embryo fibroblasts, oligodendrocytes precursor cells, and primary astrocytes [13,14]. Moreover, we showed that while Mut fibroblasts fully compensate for their faulty OXPHOS by increasing their mitochondria biogenesis, astrocytes and oligodendrocytes fail to achieve complete restoration of mitochondrial respiration per cell, despite their massive adaptation efforts which include substantial mitochondria biogenesis and increased glycolysis rates. The difference in energy requirements between fibroblasts and glial cells could explain in part the asymptomatic phenotype of fibroblasts and the high vulnerability and pathological manifestations of brain glial cells in VWMD patients [13,14,15,16].The significant roles of astrocytes, the most abundant glial cells in the brain, in all aspects of brain homeostasis including energy metabolism, prompted us to learn more about their Achilles’ heel given their excessive sensitivity to Eif2b mutations. We focused on mTORC1 and AMPK which are key antagonizing regulators of cellular energy and biomass production [17]. The mTORC1 axis stimulates anabolic pathways and ATP consumption by facilitating protein synthesis, lipogenesis, cell growth, and proliferation [17,18]. AMPK responds to energy stress by inhibiting mTOR and by enhancing catabolic pathways to stimulate ATP production mostly by promoting mitochondrial biogenesis and function and by facilitating fatty acid oxidation (FAO) [19,20,21,22,23].

In the current study, we analyzed primary astrocytes isolated from brains of Mut and WT mice. We discovered that Mut astrocytes suffer from low energy, exhibited by low ATP levels. Despite their poor energetic status, Mut astrocytes show a ROS-mediated inferior ability to stimulate the AMPK axis and favor an adaptation program that includes stimulation of mTORC1 activity, which plays a key role in redox homeostasis and allows oxidative glycolysis, similar to cancer cells. Upon further energy stress imposed by severe limitation of glucose availability, Mut astrocytes exhibit impaired adaptation capacity due to inferior ability to oxidize fatty acids. Consequently, they suffer from compromised ability to execute high ATP-demanding functions such as cell migration. The poor energetic status of Mut astrocytes and their increased vulnerability to ROS and to low glucose availability provides important additional insights to the pathology of VWMD.

## 2. Materials and Methods

### 2.1. Mice

Wild type (WT; C57BL strain) and Eif2b5^R132H/R132H^ (Mut; mutant) mice were bred and housed in Tel Aviv University animal facility with 14/10 h light/dark cycle in groups of four animals per individually ventilated cage (Lab Products Inc., Seaford, DE, USA) supplemented with autoclaved wood chips. Animals were fed with irradiated rodent hybrid pellet (#1318M, Altromin; Lage, Germany) and sterile water ad libitum. All experimental procedures were approved by the Tel Aviv University Animal Care Committee according to national guidelines (permit #04-17-022). Breeding and genotyping were performed as previously described [10].

### 2.2. Primary Astrocytes Isolation and Usage

Brains were extracted from the cranial vault of newborn (P0-P2) WT and Mut mice followed by removal of the midbrain and olfactory bulb. After meninges removal, both hemispheres were dissociated to single-cell suspension by papain digestion using MACS Neural Tissue Dissociation kit and Gentle-MACS dissociator (#130-092-628 and #130-093-235, respectively; Miltenyi Biotec, Bergisch Gladbach, Germany). Astrocytes were positively selected using anti-ACSA2 MicroBead Kit (#130-097-678 Miltenyi Biotec). A sample of selected cells was stained with GLAST (ACSA-1)-APC antibody (#130-098-803 Miltenyi Biotec) for 10 min at 4 °C in the dark, followed by flow cytometry analysis to assay purity. All isolation steps were according to the manufacturer’s protocols. ~94% purity was obtained and the yield was ~5.5 × 10^5^ astrocytes per brain. Plates used for seeding were pre-coated prior to seeding by over-night (ON) incubation at 4 °C with 0.01 mg/mL Poly-D-Lysine (PDL) (#P0899; Sigma-Aldrich, St. Louis, MO, USA) followed by 2 washes with phosphate-buffered saline (PBS). Immediately after isolation, astrocytes were seeded at a density of 5 × 10^5^ cells per PDL-pre-coated 6 cm plate and incubated in a 37 °C-5% CO_2_ incubator using ‘astrocyte medium’ (#1801; ScienCell Research Labs, Carlsbad, CA, USA) which contained astrocytes growth supplements and 2% Fetal Bovine Serum (FBS). After 7 days of incubation, cells were trypsinized with 0.25% trypsin-EDTA (#25200-072, Thermo Fisher Scientific, Waltham, MA, USA) and transferred to PDL-precoated 10 cm plate for 3 incubation days followed by a second transfer to PDL-precoated 15 cm plate for 3 additional incubation days. The cells were then trypsinized; a small sample was seeded for 48 h incubation in DMEM-HG medium for MitoTracker^®^ Deep Red FM (#M22426, Thermo Fisher Scientific) staining followed by flow cytometry analysis, while all the rest were frozen at a density of 1 × 10^6^ cells per ml per tube using freezing medium containing 90% FBS and 10% DMSO (#D5879, Sigma-Aldrich) and stored in liquid nitrogen. All experiments were performed using astrocytes at this exact stage, freshly thawed, counted, seeded and incubated with ‘astrocytes medium’ for 24 h. The medium was then changed to Dulbecco Modified Eagles Medium (DMEM) containing 2 mM L-glutamine, without glucose and sodium pyruvate (#11966025; Thermo Fisher Scientific), supplemented with 1% penicillin-streptomycin (#03-031-1B; Biological Industries, Beit Haemek, Israel), 10% FBS (#12657-029; Thermo Fisher Scientific) and glucose (G7021, Sigma-Aldrich) to final concentrations as indicated in each experiment. The ‘DMEM medium’ was used for experiments. For most experiments either DMEM supplemented with 25 mM glucose (High Glucose, DMEM-HG) or DMEM lacking glucose for the purpose of glucose starvation (DMEM-GS) was used.

### 2.3. Protein Extraction and Immunoblot Analysis

WT and Mut primary astrocytes were thawed and seeded at a density of 1 × 10^5^ astrocytes per well of a PDL-pre-coated 6-well plate for 24 h incubation in ‘astrocyte medium’ followed by further 48 h incubation in DMEM containing the indicated glucose concentration. After washing with phosphate-buffered saline (PBS), proteins were extracted using 100 μL of 2× sample buffer (4% SDS, 220 mM DTT, 20% glycerol, 180 mM Tris pH 6.8 and traces of Bromo Phenol Blue) applied directly on each monolayer. The extract, collected by a rubber policeman, was boiled and equal volumes representing equal number of cells were loaded per lane of 7, 10, or 12% SDS-polyacrylamide gels (SDS-PAGE). To analyze brain proteins, cerebrums were removed from WT and Mut mice at the age of interest, flashed frozen in liquid nitrogen and kept in −80 °C until use. Proteins were extracted by sonication, using 500 μL per hemisphere of lysis buffer [1% triton, 0.5% Na-deoxycholate, 0.1% SDS, 50 mM Tris pH 8.0, 100 mM NaCl, 10 mM β-glycerophosphate, 5 mM NaF, 1 mM DTT, 1 mM vanadate, and EDTA-free Complete^TM^ protease inhibitor cocktail (#11-836-170-001; ROCHE, Basel, Switzerland)]. Following spinning at 13,000 rpm in microcentrifuge for 15 min at 4 °C, the supernatant was kept and used for total protein concentration analysis by BCA protein assay kit (#23227 Thermo Fisher Scientific). Equal amounts of total protein were loaded per lane of 7, 10, or 12% SDS-PAGE, depending on the experiment, followed by immunoblot analyses as detailed in the Supplemental information. Briefly, the following antibodies were used. Primary: rabbit anti p-AMPK, mouse anti t-AMPK, rabbit anti p-ACC, rabbit anti t-ACC, mouse anti p-S6K, rabbit anti t-S6K, rabbit anti G6PD (#2535, #2793, #11818, #3676, #9206, #2708, #12263, respectively, Cell Signaling, Danvers, MA, USA), mouse anti mt-COI, mouse anti SDHB, rabbit anti-acetylated P53, rabbit anti-FTH1, (#ab14705, #ab14714, #ab183544, #ab75973, respectively, Abcam, Cambridge, MA, USA), mouse anti Actin (#A3853, Sigma-Aldrich). Secondary: peroxidase-conjugated goat anti mouse IgG and peroxidase-conjugated goat anti rabbit IgG (#115-035-166 and #111-035-144, respectively, Jackson Immuno Research, West Grove, PA, USA). Enhanced chemiluminescence (ECL) (#WBLUR0100, Millipore, Burlington, MA, USA) was used for detection of bands followed by capturing using AI600 Imager (Amersham, Buckinghamshire, UK) and quantification using ImageQuant TL software (GE Healthcare, Pittsburgh, PA, USA).

### 2.4. RNA Extraction and Real Time Quantitative PCR (RT-qPCR) (Detailed Version)

WT and Mut primary astrocytes were thawed and seeded at a density of 1 × 10^5^ astrocytes per well of a PDL-pre-coated 6-well plate for 24 h incubation in ‘astrocyte medium’ followed by further 48 h incubation in DMEM- HG or DMEM-GS medium. After washing with PBS, total RNA was extracted using RiboEx^TM^ Total RNA isolation solution (#301-001; GeneAll Biotechnology, Seoul, South Korea) according to the manufacturer’s protocol and used for reverse transcription using qScript^TM^ cDNA Synthesis Kit (#95047-100; Quanta Biosciences, Beverly, MA, USA). Steady state levels of TFAM, SCAF1, mt-COI, SDHB and β-Actin mRNAs were measured using SYBR-Green (PerfeCTa^®^ SYBR^®^ Green FastMix^®^, ROX™; #95073; Quanta biosciences) base reactions. The following oligonucleotide primers were used: TFAM Fwd 5′- CATTTATGTATCTGAAAGCTTCC -3′ and TFAM Rev 5′- CTCTTCCCAAGACTTCATTC -3′ for TFAM mRNA amplification; SCAF1 Fwd 5′- AAGGCTGTCCACAAGATCTGCC -3′ and SCAF1 Rev 5′- GGTAGCGCTGGACGTAGGC -3′ for SCAF1 mRNA amplification; mt-CO1 Fwd 5′- GAGCAAAAGCCCACTTCGC -3′ and mt-CO1 Rev 5′- AGTCTGAGTAGCGTCGTGGTA -3′ for mt-CO1 mRNA amplification; SDHB Fwd 5′- CGAGGTGGATCTGAATAAGTG -3′ and SDHB Rev 5′- CACAGATGCCTTCTCTACAA -3′ for SDHB mRNA amplification; β-Actin Fwd 5′- GCCTTCCTTCTTGGGTATGGAT -3′ and β-Actin Rev 5′- TTTACGGATGTCAACGTCACACT -3′ as an internal control for β-Actin mRNA amplification. Reactions were carried out for 40 cycles in StepOne Real-time PCR apparatus (Applied Biosystems, Waltham, MA, USA). Average relative quantity (RQ) of TFAM, SCAF1, mt-COI and SDHB mRNA relative to β-Actin endogenous control was calculated by the ΔΔCT method and normalized to WT control values.

### 2.5. Quantification of PGC1α Nuclear Localization

WT and Mut astrocytes were thawed and seeded at a density of 2.4 × 10^4^ per well of a 24-well plate on PDL-pre-coated 12mm coverslips and cultured for 24 h in ‘astrocyte medium’ followed by medium change to DMEM-GS for further incubation of 0, 4, 16 and 24 h. At the indicated time points cells were rinsed with PBS and fixed with 4% paraformaldehyde for 10 min, permeabilized with 0.1% Triton X-100 for 10 min, blocked with 4% bovine serum albumin (BSA) for 1 h and incubated with anti-PGC1α antibody (#ab54481, Abcam diluted 1:1000 in 4% BSA) for 1 h, all at RT. All solutions were prepared in PBS. Coverslips were rinsed with PBS and incubated with 1:10,000 dilution of Alexa Fluor 488- anti-rabbit IgG (A11034, Invitrogen, Waltham, MA, USA) for 2 h in the dark. Following PBS rinse, the cytoplasm and nucleus were stained with 1:1000 dilution of Solution 21 (BlueMask-1™, ChemoMetec, Lillerod, Denmark) and 1:250 dilution of solution 12 (DAPI, ChemoMetec), respectively, for 30 min in the dark. All procedures were performed at RT. Coverslips were rinsed with PBS and mounted on Xcyto 2-sample slides type 15-A (ChemoMetec), and analyzed using quantitative cell imager cytometer Xcyto-5 (ChemoMetec) at 20× magnification with excitation/filter sets AF488 (LED488; 513-555), MASK (LED405; 430-475) DAPI (LED405; 573-613). Similarity scores were calculated using XcytoView (ChemoMetec) and represent the log-transformed Pearson’s correlation coefficients between PGC1α (AF488) and DNA (DAPI) channels. Similarity scores report about the relative nuclear localization of PGC1α. ~3 × 10^3^ cells were analyzed for each experimental condition. Statistical analysis was performed using Kruskal–Wallis One-way ANOVA followed by Dunn’s test for multiple comparisons. In parallel, coverslips were stained with PGC1α antibodies and Hoechst for imaging at 20× magnification using Nikon eclipse 50i fluorescent microscope equipped with Nikon DS-QiMC camera.

### 2.6. Mitochondrial DNA (mtDNA) Quantification

WT and Mut primary astrocytes were thawed and seeded at a density of 3 × 10^4^ cells per well of a PDL pre-coated 24-well plate for 24 h incubation in ‘astrocyte medium’ followed by further 48 h incubation in DMEM-HG or DMEM-GS medium. mtDNA was quantified as detailed in [13].

### 2.7. Oxygen Consumption and Glycolytic Proton Efflux Rates

WT and Mut primary astrocytes were thawed and seeded at a density of 5 × 10^3^ astrocytes per well of a PDL-pre-coated XF96-well cell culture microplate plate (Seahorse Bioscience, Billerica, MA, USA) for 24 h incubation in ‘astrocyte medium’ followed by further 48 h incubation in DMEM-HG or DMEM-GS medium. Oxygen consumption rate (OCR) was measured using the Mito Stress Test Kit (Agilent Technologies, Santa Clara, CA, USA) as detailed in [13] using ‘seahorse medium’ (Bicarbonate-free DMEM (#102353; Agilent Technologies, Santa Clara, CA, USA) supplemented with energy source as indicated. For single fuels tests, the ‘seahorse medium’ contained either 10 mM glucose or 2 mM glutamine. To test fatty acids as a single fuel, the XF Palmitate-BSA FAO Substrate kit (Agilent Technologies) was used.

Glycolytic Proton Efflux Rate (GlycoPER) was determined using the Seahorse XF Glycolytic Rate Assay Kit (Agilent Technologies, Santa Clara, CA, USA). Rotenone and Antimycin (0.5 μM each), and 2-deoxy-D-glucose (2-DG; 50 mM) were sequentially added. All data were normalized to biomass, obtained by Crystal Violet, widely used to report cell mass for normalization purposes [24].

### 2.8. ATP Measurements

WT and Mut primary astrocytes were thawed and seeded at a density of 5 × 10^3^ cells per well of a PDL pre-coated 96-well plate for 24 h incubation in ‘astrocyte medium’ followed by further 48 h incubation in DMEM-HG or DMEM-GS medium. The ADP/ATP Ratio Bioluminescent Assay Kit (#ab65313; Abcam) was used to measure ATP levels according to the manufacturer’s instructions, employing Veritas microplate luminometer (Turner BioSystems/Promega, Madison, WI, USA). ATP levels were normalized to biomass, obtained by Crystal Violet staining [24].

### 2.9. Assessment of Cytoplasmic ROS and Cell Death by Flow Cytometry

WT and Mut primary astrocytes were thawed and seeded at a density of 1 × 10^5^ per well of a PDL pre-coated 6 well plate in ‘astrocyte medium’ for 24 h incubation followed by further 48 h incubation in DMEM-HG or DMEM-GS. For cellular ROS assessment the cells were washed and then stained for 30 min in a similar serum-free medium containing 2 μM DCF-DA (#35845; Sigma-Aldrich) followed by trypsinization and resuspension in 200 μL of PBS for flow cytometry. For apoptosis assessment both adherent and floating (dead) cells were collected and combined for staining with Annexin-V antibodies and propidium iodide (PI) using the MEBCYTO^®^ Apoptosis Kit (#4700, MBL International Corp., Woburn, MA, USA), as recommended by the supplier. In total, 5 × 10^3^ of stained cells were used for flow cytometry analysis using Stratedigm S1000EXi Flow Cytometer and CellCapTure software. Cell debris were excluded from the analysis based on the scatter signals. The FlowJo software (FLOWJO, LLC, Ashland, OR, USA) was used for data analysis and generation of histograms.

### 2.10. Wound-Scratch Assay for Migration Analysis

WT and Mut primary astrocytes were thawed and seeded at a density of 5 × 10^3^ cells per well of a PDL pre-coated 96-well plate in ‘astrocyte medium’ for 24 h followed by 72 h incubation with DMEM-GS medium until spatially uniform monolayers were formed. Uniform and reproducible scratches in each well were performed using a WoundMaker™ (Essen BioScience, Ann Arbor, MI, USA) followed by medium removal and washing with PBS to remove detached cells from the scratched area. Then, 150 μL of DMEM-GS medium with or without 4 μM of Etomoxir were added to each well and the plates were placed in an IncuCyte ZOOM™ apparatus (Sartorius, Goettingen, Germany). Images of the collective cell spreading were recorded every 2 h for a total duration of 48 h. Cell migration was analyzed by measuring the ‘wound confluence (%)’ as parameter of wound scratch recovery, using the IncuCyte ZOOM™ Scratch Wound Processing Software.

### 2.11. Statistics

For each experiment, ≥3 biological repeats were used, each containing different WT and Mut batches of astrocytes isolated from independent litters. ≥3 technical replicates were used for each biological repeat, except for Western blot (WB) and FACS analyses. Due to technical variation between different batches and between experiments, the data of each experiment was normalized to WT untreated samples, which were assigned the mean value of 1. When possible (seahorse and qPCR, where all biological and technical replicates can be analyzed simultaneously on the same plate), each data value was normalized to the average of all WT replicates, to reflect the distribution within the WT samples. For special cases such as ROS analysis and apoptosis detection (which yield highly variable baseline values across experiments and batches), the differential response of each genotype was analyzed separately. Statistical analysis was performed with Prism 9.0.1 software (GraphPad). Normal distribution was confirmed by Shapiro–Wilk test. Unpaired two-tailed Student’s T-test (after F-test for variance check) was used to compare between 2 groups. For comparisons between 3 groups, one-way ANOVA followed by Tukey’s multiple comparisons test was used for experiments with one independent variable (except for Figure 2B in which the data does not fit normal distribution and therefore Kruskal–Wallis One-way ANOVA followed by Dunn’s multiple comparison test was performed), or two-way ANOVA followed by Sidak’s multiple comparison test for experiments with two independent variables. Data are presented as mean ± SEM. * *p* ≤ 0.05, ** *p* ≤ 0.01, *** *p* ≤ 0.001, **** *p* ≤ 0.0001.

### 2.12. Compounds Used (Stocks)

Arsenite (106277, Sigma-Aldrich; 50 mM in H_2_O), AICAR (9944, Cell Signaling; 75 mM in H_2_O), 4EGI (324517, Sigma-Aldrich; 100 mM in DMSO), N-Acetyl-Cysteine (A9165, Sigma-Aldrich; freshly prepared 100 mM in H_2_O), Etomoxir (E1905, Sigma-Aldrich; 10 mM in H_2_O).

## 3. Results

### 3.1. Eif2b5^R132H/R132H^ (Mut) Astrocytes Exhibit Low AMPK Activity Despite Low Energy Status

**Figure 1:**
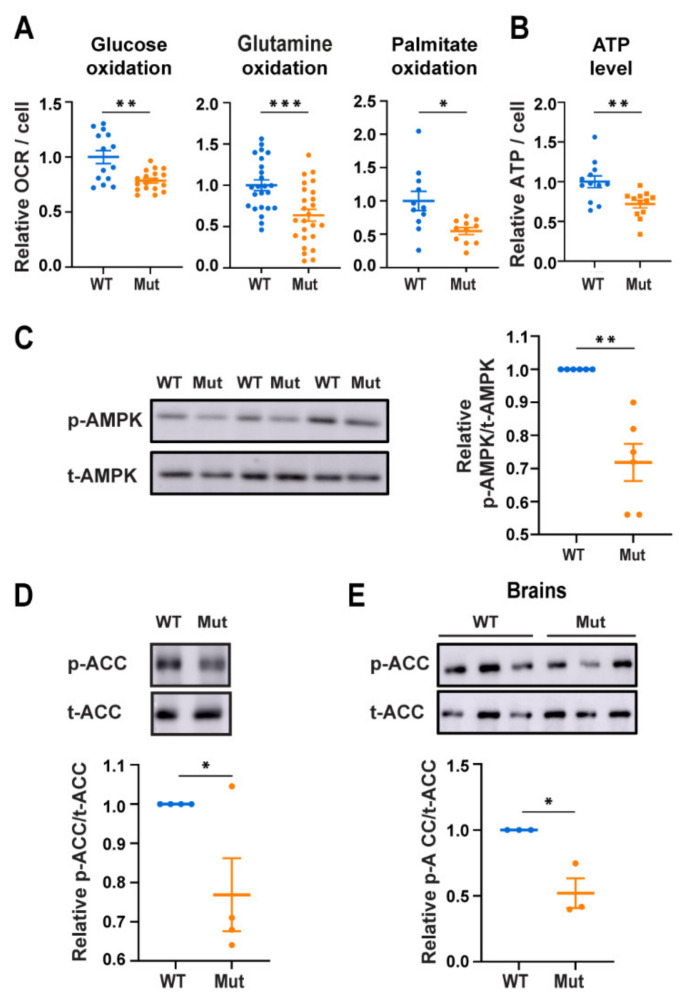
Oxidative respiration, ATP level and AMPK activity in WT (blue) and Mut (orange) primary astrocytes. (**A**) Basal respiration in the presence of glucose, glutamine or palmitate as a single fuel. Values represent oxygen consumption rate (OCR) per cell normalized to the mean value of WT. (**B**) Level of ATP per cell in astrocytes grown in DMEM-HG normalized to the mean value of WT. (**C**) Immunoblot analyses of equal amounts of total protein of astrocytes grown in DMEM-HG, using antibodies specific for phospho-AMPK (p-AMPK) and total-AMPK (t-AMPK). (**D**,**E**) Immunoblot analyses of equal amounts of total protein of astrocytes grown in DMEM-HG, or brains at postnatal age of 10 months, using antibodies specific for phospho-ACC (p-ACC) and total-ACC (t-ACC). Representative blots are shown. Graphs present average values ± SEM relative to WT. Statistical analysis is detailed in Materials and Methods. * *p* ≤ 0.05, ** *p* ≤ 0.01, *** *p* ≤ 0.001.

To reach maximal experimental consistency we used cultures highly enriched (>94%) with ACSA1-positive astrocytes, isolated by affinity columns from brains of WT and Mut newborns (Supplementary Figure S1). The anomalous phenotype of Mut astrocytes [13] was confirmed by MitoTracker staining (Supplementary Figure S2). Since astrocytes are capable of using various fuels for generation of respiration-derived ATP, we first tested if the respiration deficit of Mut [12,13] is a general deficit rather than fuel-specific. For this purpose, glucose, glutamine and palmitate as single energy sources were used as test cases. We found that Mut astrocytes exhibit 21%, 36% and 45% lower oxidation efficiency of each of these fuels, respectively, compared to WT (Figure 1A). This finding supports the notion of a general OXPHOS impairment, probably due to the compromised composition and performance of Mut ETC complexes [12]. In agreement with their deficit, Mut astrocytes exhibit a 28% decrease in ATP level compared to WT (Figure 1B; *p* < 0.01). To further confirm the inborn poor energetic status, we assessed the activity of AMPK, the energy homeostasis key regulator. Surprisingly and counterintuitively, we found a reduction of 28% in phospho-AMPK (p-AMPK) per total-AMPK (t-AMPK) in Mut compared to WT (Figure 1C; *p* = 0.004) despite their lower ATP level. Moreover, the phosphorylation status of acetyl-CoA carboxylase (ACC), one of AMPK downstream targets, was lower by 23% and 48%, in Mut astrocytes and lysates of brains isolated from 10-month-old Mut mice, respectively, compared to WT controls (Figure 1D,E). These findings show that AMPK activity is paradoxically lower in primary astrocytes and whole brain tissues expressing mutated eIF2B, despite their poor energetic status.

### 3.2. Eif2b5^R132H/R132H^ (Mut) Astrocytes Exhibit Limited Adaptation to Energy Stress

**Figure 2:**
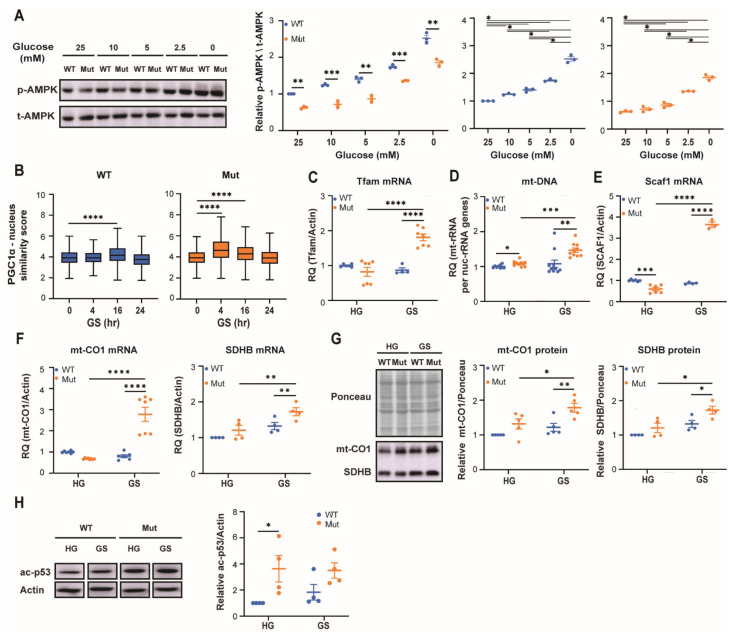
Impact of energy stress on AMPK activity and its downstream effector in WT (blue) and Mut (orange) primary astrocytes. (**A**) Immunoblot analysis of p-AMPK and t-AMPK following 48 h incubation with the indicated glucose concentrations. (**B**) PGC1α nuclear localization represented by boxplots showing the median, minimum and maximum similarity scores of PGC1α and DAPI fluorescence signals obtained from 3 × 10^3^ cells, analyzed at each of the indicated time points along glucose starvation. Representative images shown in Supplementary Figure S3. (**C**,**E**,**F**) RT-qPCR analysis of Tfam, Scaf-1, mt-CO1 and SDHB per actin mRNA levels following 48 h incubation in DMEM-HG or DMEM-GS medium. Data represent average of RQ values per actin mRNA. (**D**) Quantification of mitochondrial DNA (mtDNA) following 48 h incubation in DMEM-HG or DMEM-GS medium. Data represent average of RQ values of DNA encoding mitochondrial 12S rRNA per nuclear 18S rRNA genes. (**G**) Immunoblot analysis of mt-COI and SDHB protein levels following 48 h incubation in DMEM-HG or DMEM-GS medium. Data represent average of the ratio of mt-CO1 or SDHB band intensity per total protein in the lane as quantified by Ponceau staining. (**H**) Immunoblot analysis of acetylated-p53 (ac-p53) and actin protein levels following 48 h incubation in DMEM-HG or DMEM-GS medium. Data represent average of ac-p53 per actin ratio. Representative blots are shown. All graphs except for B show the average values ± SEM relative to WT-HG. Statistical analysis is detailed in Materials and Methods. * *p* ≤ 0.05, ** *p* ≤ 0.01, *** *p* ≤ 0.001, **** *p* ≤ 0.0001.

**Figure 3:**
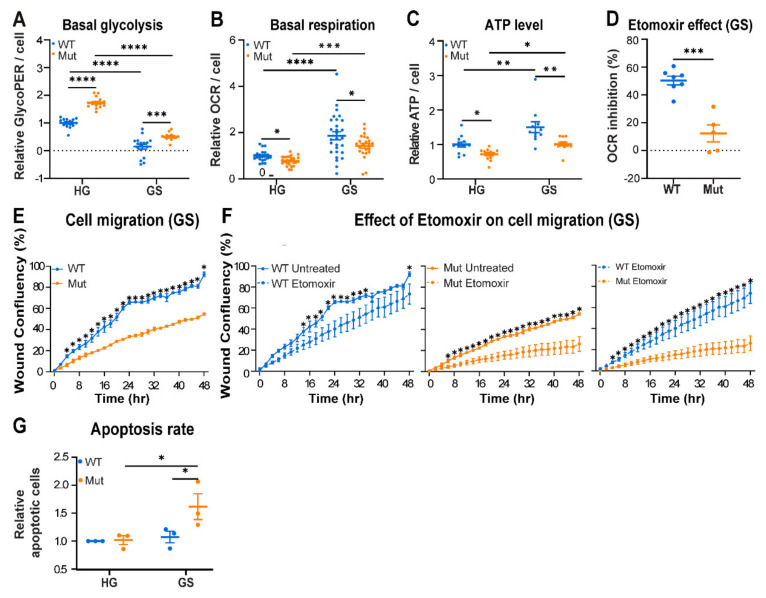
Impact of glucose starvation (GS) on energy status and function of WT (blue) and Mut (orange) primary astrocytes. Cells were incubated for 48 h in DMEM-HG or DMEM-GS medium followed by analyses of: (**A**) Basal glycolysis. Data represent glycolytic proton efflux rate (GlycoPER) per cell. (**B**) Basal respiration. Data represent OCR per cell. (**C**) ATP level per cell. Values in ABC are relative to the mean value of WT-HG. (**D**) Astrocytes grown in DMEM-GS for 48 h were pre-treated or not with 4 µM Etomoxir for 15 min and analyzed for basal respiration using ‘seahorse medium’ supplemented with 10% FBS as fatty acids source, with or without 4 µM Etomoxir, respectively. Data show % OCR inhibition by Etomoxir. (**E**,**F**) Cell migration by wound-scratch assay along 48 h of incubation in DMEM-GS using IncuCyte ZOOM™ apparatus, in the presence or absence of 4 µM Etomoxir. Shown is average of % wound confluency recorded at 2 h intervals. (**G**) Cell death assessed by staining with FITC-conjugated Annexin-V antibodies followed by analysis by flow cytometry. Data represent average ± SEM of fold change Annexin-V positive cells relative to WT HG. Statistical analysis is detailed in Materials and Methods. All graphs show the average values ± SEM. * *p* ≤ 0.05, ** *p* ≤ 0.01, *** *p* ≤ 0.001, **** *p* ≤ 0.0001.

To test if the lower AMPK activity in Mut astrocytes is due to defective energy sensing, we exposed them for 48 h to different concentrations of glucose, which is the prefered fuel used by astrocytes for energy production. The decrease in glucose concentrations revealed a corresponding increase in AMPK activity by both genotypes. However, under all glucose concentrations, AMPK activity was always significantly lower by 22–46% in Mut compared to WT (Figure 2A). Assuming that astrocytes store glycogen, we exposed them to glucose starvation (GS) conditions for 48 h to achieve further decrease in glucose availability and then assessed adaptation events downstream of AMPK compared to cells grown in high glucose (HG, 25 mM) medium. We first looked at peroxisome proliferator-activated receptor gamma coactivator 1-α (PGC-1α), a key controller of mitochondrial function and biogenesis. Active PGC-1α is known to transiently accumulate in the nucleus where it supports expression of genes encoding transcription factors required for enhancement of mitochondria functions when cells face energy deprivation [25,26]. The data reveal a faster response of Mut astrocytes to GS, as judged by the faster nuclear accumulation of PGC-1α in their nuclei. Specifically, Figure 2B shows 20% increase in nuclear PGC-1α level after 4 h, only in Mut, compared to a slighter increase of 7.5% (*p* < 0.0001), after 16 h in WT astrocytes. Moreover, despite the faster nuclear accumulation of PGC-1α in Mut astrocytes, it returned to basal nuclear level only after 24 h, probably following sufficient expression of its target genes (Figure 2B and Supplementary Figure S3). One of the factors induced by PGC-1α is mitochondrial transcription factor A (Tfam) which drives transcription and replication of mtDNA to support the formation of additional ETC complexes [27]. Figure 2C,D shows 2.2- and 1.35-fold GS-mediated increase in the level of Tfam mRNA and mtDNA, respectively, only in Mut astrocytes (*p* < 0.001), reflecting their adaptive response and indicating their hypersensitivity to energy stress. An additional indication is the 6-fold increase (*p* < 0.0001) in supercomplex assembly factor 1 (*Scaf1*) expression, only in Mut astrocytes (Figure 2E). Scaf1 is known to induce the formation of supercomplexes in order to modulate OXPHOS efficiency by optimizing electron flux [28,29]. Generation of ETC complexes requires coordinated expression from both mitochondrial and nuclear genomes. Therefore, to validate actual increase in formation of ETC complexes we quantified the expression levels of mtCO1, a mitochondria-encoded catalytic subunit of ETC complex IV, and SDHB, a nuclear-encoded subunit of ETC complex II. We found that in response to GS conditions, Mut and not WT astrocytes exhibit an increase in expression of mtCO1 and SDHB genes at both the mRNA and protein levels (Figure 2F,G). Collectively, despite their lower AMPK activity, Mut astrocytes present increased activation of PGC-1α-related pathways. While this could be due to their faster response to energy deprivation (Figure 2B), activation of alternative upstream regulatory pathway that do not involve AMPK, such as the p38 MAPK-mediated stress response [30], can not be ruled out. To further understand the phenomenon of compromised AMPK activity in Mut astrocytes, we set to look at another arm downstream of AMPK, designed to combat reactive oxygen species (ROS) which accompanies the increase in OXPHOS and changes cellular redox state. Such a redox regulator is the NAD-dependent deacetylase sirtuin-1 (SIRT1) which induces the expression of antioxidant genes [20]. To assess SIRT1 enzymatic activity we monitored the level of acetylated p53 (ac-p53), one of its many direct targets. We found that ac-p53 level was ~3.5-fold higher in Mut compared to WT when cultured in high glucose (Figure 2H), implying a significant decrease in SIRT1 deacetylase activity in Mut astrocytes. This specific finding agrees with the lower AMPK activity in Mut cells (Figure 1 and Figure 2). Together, these results show (i) the unexpected requirement of Mut astrocytes for a lower level of AMPK activity; and (ii) the higher dependence of Mut astrocytes on activation of an alternative adaptive response that enables increased mitochondrial OXPHOS, emphasizing their vulnerability to energy stress. Next, we explored the functional consequences of the anomalous adaptation of Mut astrocytes to energy stress. The 1.7-fold increase in glycolysis rate in Mut compared to WT under high glucose conditions indicates that glycolysis is part of their adaptation program to the inborn OXPHOS deficit (Figure 3A; (*p* < 0.0001). Although 48 h of glucose starvation has led obviously to a marked decrease in glycolysis, it did not completely abolish it, probably due to glycogen stores (Figure 3A). In the absence of glucose it is expected that AMPK will promote respiration by fatty acids beta-oxidation (FAO) to maintain cellular survival, as mitochondrial FAO yields higher ATP level compared to glucose oxidation. However, while GS triggered ~1.8-fold increase in OCR in both WT and Mut astrocytes (Figure 3B), ATP in Mut did not reach WT level (Figure 3C), despite the extensive adaptive response shown in Figure 2. To confirm a shift to FAO upon GS, we tested the effect of Etomoxir, an inhibitor of fatty acids transport into the mitochondria. In the presence of high glucose, neither WT nor Mut astrocytes obtained energy through FAO (not shown). However, upon GS both shifted to FAO, but Etomoxir inhibited OCR by 50% in WT astrocytes and only by 12% in Mut astrocytes (Figure 3D). This finding suggests that Mut are less productive in using FAO than WT astrocytes, which could explain their lower ATP level. To assess possible functional consequences, we evaluated cell migration, a high energy-consumig process. To this end, confluent monolayers under GS conditions were subjected to a wound scratch assay, in the presence or absence of Etomoxir. We found that (i) under GS conditions Mut astrocytes migrate less efficiently than WT (Figure 3E); and (ii) Etomoxir caused a greater inhibitory effect on cell migration of Mut compared to WT astrocytes (Figure 3F). This outcome demonstrates that Mut astrocytes are more dependent on FAO than WT for execution of high-energy demanding physiological tasks, but they use it less efficiently (Figure 3D). To further evaluate the vulnerability of Mut astrocytes to energy stress, their death rate upon 48h GS was assessed by flow cytometry, showing a 1.6-fold increase (*p* = 0.035) in the fraction of annexin-positive (apoptotic) Mut astrocytes compared to WT (Figure 3G). Although the dying sub-population is small, it represents the hyper sensitivity of Mut astrocytes. These findings indicate that although under energy stress conditions Mut astrocytes promote generation of effectors to enhance mitochondrial respiration (Figure 2B–G), they fail to promote sufficient AMPK activity and consequent FAO. Due to their inability to generate sufficient level of ATP they suffer functional consequences.

### 3.3. Eif2b5^R132H/R132H^ (Mut) Astrocytes Are Hypersensitive to ROS

**Figure 4:**
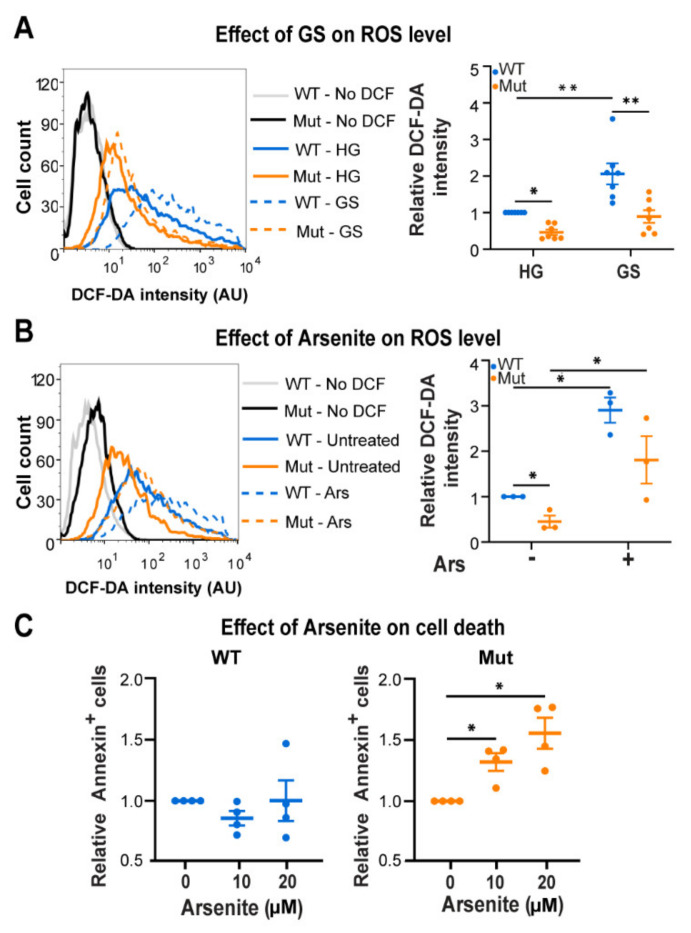
GS connection to ROS and the effect of ROS on WT (blue) and Mut (orange) primary astrocytes. Cells were incubated for 48 h with either DMEM-HG or DMEM-GS medium (**A**), or with DMEM-HG followed by 4h treatment with 20 μM Ars (**B**), or for 24 h with 10 or 20 μM Ars (**C**). Cells were then stained with DCF-DA (**A**,**B**) or with FITC-conjugated Annexin-V antibodies (**C**) followed by flow cytometry analysis. Left panels show representative histograms. Right panels show the mean fluorescence intensity of DCF-DA relative to WT-HG (**A**,**B**) or fold change of Annexin-V positive cells relative to WT-HG (**C**). All Data represent the average values ± SEM. Statistical analysis is detailed in Materials and Methods. * *p* ≤ 0.05, ** *p* ≤ 0.01.

**Figure 5:**
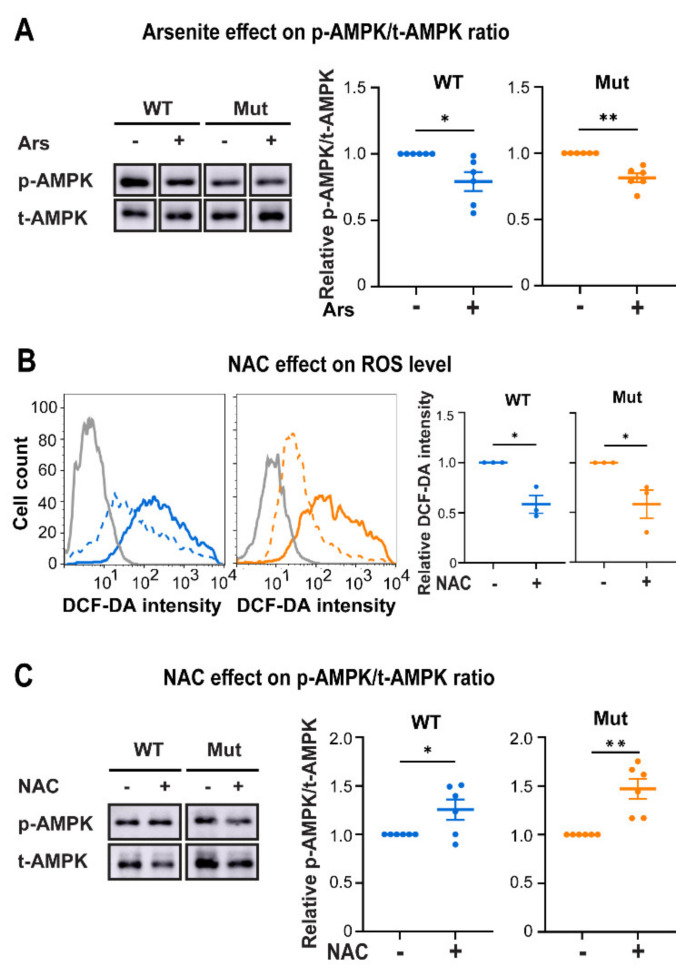
Effect of Ars and NAC on ROS level and AMPK activity in WT (blue) and Mut (orange) primary astrocytes. Cells were cultured for 48 h in DMEM-HG medium in the presence or absence of 20 µM Ars for the last 24 h (**A**) or in the presence or absence of 4 mM N-Acetyl-Cysteine (NAC) for 48 h (**B**,**C**) followed by immunoblot analyses using antibodies specific for p-AMPK and t-AMPK. Representative blots are shown; data represent average ± SEM of p-AMPK/t-AMPK ratio relative to untreated samples (**A**,**C**). Flow cytometry analysis after DCF-DA staining is shown in B. Grey, unstained; Blue, WT; Orange, Mut (continuous lines, untreated; dotted lines, NAC-treated). Graph presents the average ± SEM of mean DCF-DA fluorescence intensity relative to untreated cells. Statistical analysis is detailed in Materials and Methods. * *p* ≤ 0.05, ** *p* ≤ 0.01.

We next hypothesized that the limited adaptive capacity of Mut astrocytes to energy stress might be due to a constraint they are facing. Since SIRT1 is a redox regulator, its low enzymatic activity in Mut astrocytes (Figure 2H) prompted us to assess cellular ROS levels. To look into this question we used DCF-DA staining and found ~2-fold increase in ROS level upon GS in both genotypes (Figure 4A). However, the observed absolute ROS levels unveiled an unexpected phenomenon. Counterintuitively, Mut astrocytes exhibited 60% decrease in ROS level compared to WT under both HG and GS conditions (Figure 4A). Exposure to Arsenite (Ars), an effective ROS-generating agent, caused 2.9-fold and 4.0-fold increase in ROS level in WT and Mut, respectively; but again, Mut astrocytes exhibited lower level of ROS compared to WT under all conditions (Figure 4B). Moreover, the Ars treatment has led to a 1.3–1.5-fold (*p* < 0.05) increase in the level of Annexin-positive cells only in Mut (Figure 4C), similar to the increase in apoptosis rate of Mut astrocytes under GS conditions (Figure 3G). These observations indicate that compared to WT, Mut astrocytes (i) harbor low cellular ROS levels; and (ii) are more sensitive to ROS. Such a phenomenon can be attributed to either slower ROS production or faster ROS clearance in Mut astrocytes. The low ROS level in Mut even upon Ars treatment favors the possibility of a faster ROS scavenging capacity (Figure 4B). Their low SIRT1 and AMPK activities brings up the possibility that Mut astrocytes execute an alternative signaling axis for antioxidants production which is not fully compatible with the WT signaling pathways. To clarify the relationships between ROS level and AMPK activity in astrocytes, we used Ars or N-acetyl-L-cystein (NAC) to promote or abolish ROS, respectively. Whereas Ars treatment triggered a 20% decrease in *p*-AMPK/t-AMPK ratio in both genotypes (Figure 5A, *p* < 0.02), NAC treatment led to 40% decrease in ROS (Figure 5B, *p* < 0.04) and prompted a 1.26- and 1.47-fold increase in p-AMPK/t-AMPK ratio in WT and Mut, respectively (Figure 5C, *p* < 0.04). These experiments provided the indication that high ROS in astrocytes is not compatible with high AMPK activity. This surprising notion that low AMPK activity in astrocytes is in fact a proxy of extensive ROS generation, actually conflicted with the information that Mut astrocytes present low AMPK activity and low ROS levels (Figure 1C, Figure 2A and Figure 4A,B). A possible justification for this oxymoron might be the existence of an efficient ROS scavenging system in Mut astrocytes, via an alternative antioxidants-generating pathway that is incompatible with AMPK activity and acts at a faster rate than the ROS accumulation rate in these cells. In accord with this view, it is possible that NAC lowers ROS to a level that makes the alternative pathway worthless, thus allowing higher AMPK activity in Mut astrocytes (Figure 5B,C).

### 3.4. Eif2b5^R132H/R132H^ (Mut) Astrocytes Employ Higher mTORC1 Activity for Redox Regulation

**Figure 6:**
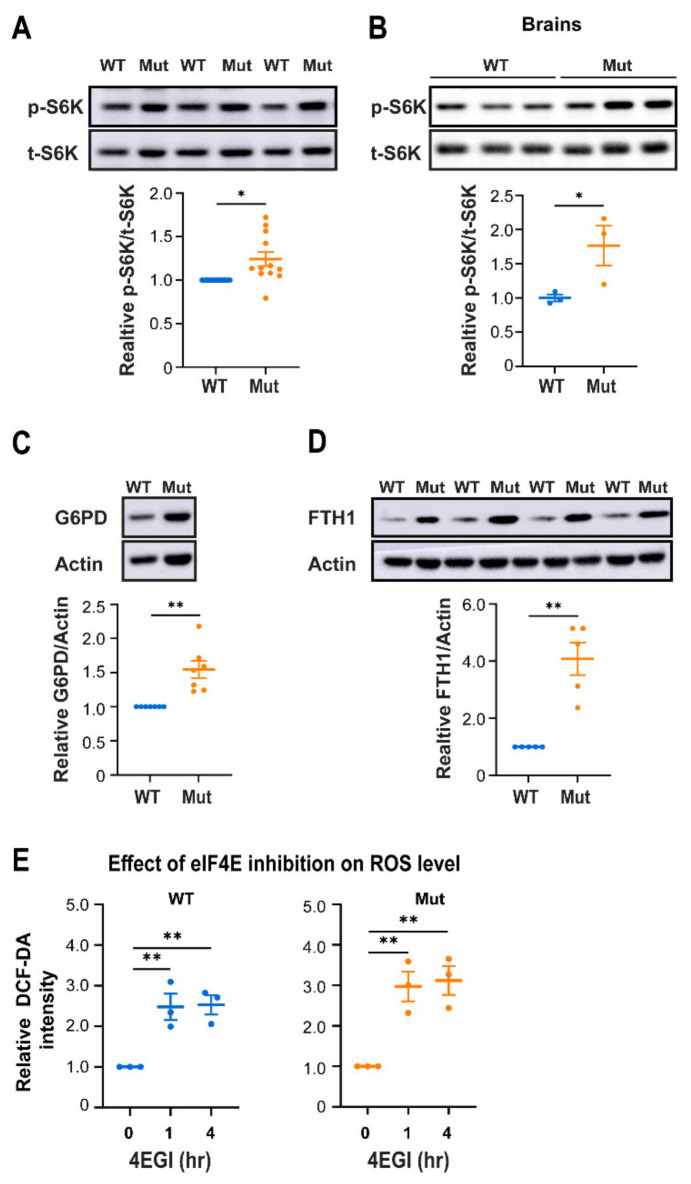
mTORC1 activity and its implications in WT (blue) and Mut (orange) primary astrocytes. (**A**–**D**) Equal amounts of total protein of primary astrocytes cultured in DMEM-HG, or brains at postnatal age of 10 months, were subjected to immunoblot analysis using antibodies specific for phospho-S6K (p-S6K) and total-S6K (t-S6K) (**A**,**B**), or G6PD and actin (**C**), or FTH1 and actin (**D**). Representative blots are shown. Data represent the average of the indicated ratios ± SEM normalized to WT. (**E**) Primary astrocytes grown in DMEM-HG were treated or not for 1 or 4 h with 400 µM of 4EGI prior to analysis of ROS level by DCF-DA staining and flow cytometry. Data represent the average values of mean DCF-DA intensity ± SEM normalized to untreated cells. Statistical analysis is detailed in Materials and Methods. * *p* ≤ 0.05, ** *p* ≤ 0.01.

To further explore this possibility, we hypothesized that Mut astrocytes continuously produce high level of ROS which is instantaneously scavenged by a constant supply of ROS-regulating proteins that are critical for Mut survival. Inspired by cancer cells which constantly face the challenge of high ROS level and thereby generate antioxidant proteins downstream of mTORC1 signaling in order to combat it [31], we set out to test mTORC1 activity by measuring the phosphorylation status of rpS6 kinase (S6K), one of its direct targets. Interstingly, we found 1.24- and 1.77-fold increases in the p-S6K/t-S6K ratio in Mut primary astrocytes (Figure 6A) and lysates of brains isolated from 10 month-old mice (Figure 6B), respectively, compared to WT controls. To verify the expected increase in the expression of mTORC1-induced antioxidants in Mut astrocytes, we chose to assess the protein levels of two relevant representatives: (i) glucose-6-phosphate dehydrogenase (G6PD), the rate limiting enzyme in the pentose phosphate pathway (PPP); and (ii) Ferritin heavy chain 1 (FTH1), a ferroxidase important for iron homeostasis [32]. While PPP generates NADPH for maintenance of reduced glutathione required for ROS neutralization [33], FTH1 function protects from the toxic effects of ROS generated by iron in the presence of oxygen when iron-sulfur (FeS) clusters accumulate upon induction of ETC complexes formation [34]. We found 1.5- and 4.1-fold increase in the level of G6PD and FTH1 proteins, respectively, in Mut compared to WT astrocytes (Figure 6C,D). Remarkably, GS caused a marked increase in FTH1 protein level only in WT astrocytes, most probably due to the increase in ROS level upon GS, but not in Mut astrocytes which exhibit high FTH1 level already before GS (Supplementary Figure S4), again indicating the inborn anomalous phenotype of astrocytes due to R132H mutation in Eif2b5.

Importantly, the activity of translation initiation factor eIF4E is directly regulated by mTORC1, while a subset of mRNAs encoding ROS-regulating proteins including FTH1 depend for their translation on high dose of active eIF4E [35,36]. To substantiate the link between eIF4E-dependent translation (a proxy of mTORC1 activity) and cytoplasmic ROS levels, we tested the effect of 4EGI-1, a specific inhibitor of eIF4E-eIF4G interaction [37], on ROS level. Treatment for 1 h with 400 µM 4EGI-1 has led to 2.5- and 3.0-fold increase in ROS levels in WT and Mut astrocytes, respectively (Figure 6E). These observations agree with dependence of astrocytes on a specific subclass of ROS-regulating proteins downstream of mTORC1. Given that AMPK and mTOR activities antagozize each other, it also provides explanation for the lower AMPK activity in Mut compared to WT astrocytes.

**Figure 7:**
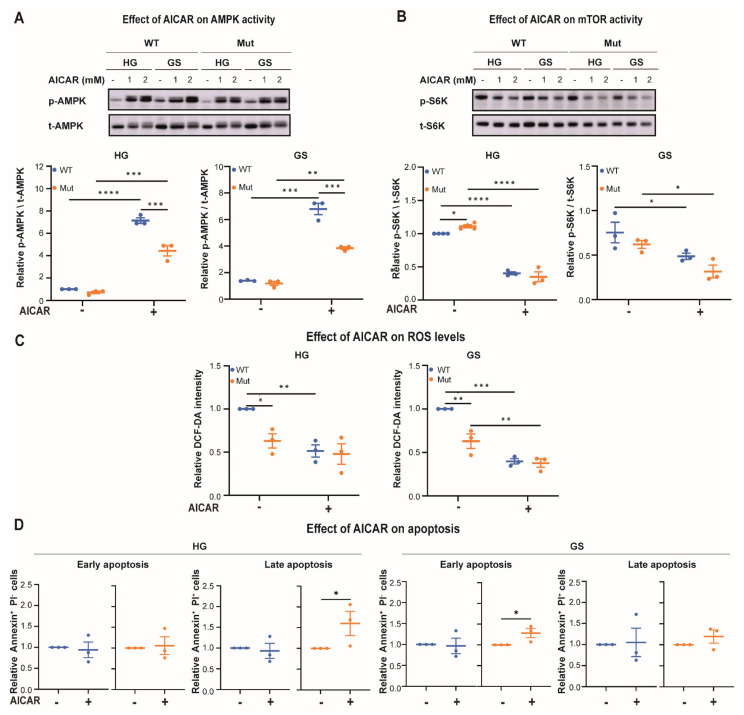
Effect of AICAR on WT (blue) and Mut (orange) primary astrocytes. (**A**,**B**) Cells were grown for 48 h in DMEM-HG or DMEM-GS medium and treated or not for the last two hours with 1 or 2 mM AICAR followed by analysis. (**A**,**B**) Total protein extracts were subjected to immunoblot analyses using antibodies for p-AMPK and t-AMPK (**A**), or p-S6K and t-S6K (**B**). Representative blots are shown. Data represent the effect of 2 mM AICAR and show the average of the indicated ratios ± SEM normalized to untreated WT-HG. (**C**,**D**) Cells were stained with DCF-DA (**C**), or FITC-conjugated Annexin-V antibodies and propidium iodide (PI) (**D**), followed by flow cytometry analyses for the detection of ROS level and cell death, respectively. Data represent average ± SEM of mean DCF-DA fluorescence intensity relative to WT (**C**), or fold change of Annexin positive|PI negative or Annexin positive|PI positive cells, relative to untreated controls (**D**). Statistical analysis is detailed in Materials and Methods. * *p* ≤ 0.05, ** *p* ≤ 0.01, *** *p* ≤ 0.001, **** *p* ≤ 0.0001.

To further challenge the notion that Mut astrocytes can not tollerate high AMPK activity and require mTORC1 activity for redox regulation, we tested the effect of 2 h treatment with AICAR, a potent AMPK activator. Under normal HG conditions, AICAR led to 7.1- and 4.4-fold increase in p-AMPK/t-AMPK ratio (Figure 7A, HG; *p* < 0.001), accompanied by 60% and 65% decrease in mTORC1 activity (Figure 7B, HG; *p* < 0.0001) in WT and Mut, respectively. This treatment casued 49% reduction in ROS levels in WT and brought it to Mut level (Figure 7C, HG; *p* < 0.01), demonstrating the ability of the AMPK axis to scavenge ROS in WT, but not in Mut astorocytes. However, while AICAR-mediated AMPK activation was not harmful for WT astrocytes, it led to 1.6-fold increase in the fraction of late apoptotic Mut astrocytes population, implying that 2 h of vigorous AMPK activation under HG conditions is toxic for Mut, but not for WT (Figure 7D, HG). This finding supports the notion that Mut astrocytes can not bear high AMPK activity under normal conditions, presumably because they require a different set of antioxidats in order to survive. To further confirm the higher vulnerability of Mut astrocytes to ‘non-accurate’ AMPK activation upon energy stress, we also assessed the effect of AICAR treatment during the last 2 h of 48 h glucose starvation treatment. We found that it led to 6.9- and 3.3-fold increase in AMPK activity (Figure 7A, GS; *p* ≤ 0.01), accompanied by 35% and 48% reduction in mTORC1 activity (Figure 7B, GS; *p* < 0.05) and 60% and 40% reduction in ROS levels (Figure 7C, GS; *p* < 0.01) in WT and Mut, respectively. However, while it did not affect the viability of WT astrocytes, it led to 1.3-fold increase in the early apoptotic population of Mut astrocytes (Figure 7D, GS; *p* = 0.03). The data, which reflect a snapshot at a specific time point (i.e., 2 h of AICAR treatment), show that under HG conditions a sub-population of Mut astrocytes is already in late apoptosis, while WT astrocytes are still healthy and do not show any signs of death, even not early apoptosis. However, when AICAR is present under GS conditions which are harmful for Mut astrocytes, the most sensitive sub-population of Mut is probably already gone while the next sub-population of Mut astrocytes experience early apoptosis and head towards their apoptotic death (Figure 7D). The data further emphasize the hypersensitivity of Mut astrocytes to high AMPK activity.

## 4. Discussion

We reported earlier that increase in mitochondria biogenesis (monitored by assessment of mtDNA content), is a powerful adaptation strategy of eIF2B-mutant fibroblasts, as it fully compensates their OXPHOS deficit. In contrast to Mut fibroblasts which exhibit elevated levels of mtDNA and ROS together with normal levels of ATP per cell [13], the substantial increase in mitochondria components in Mut astrocytes does not yield sufficient production of ATP (Figure 1B and Figure 3C, Supplementary Figure S2). Possibly, fibroblasts do not require high energy for their functions, thus the increase in OXPHOS required to meet their needs is within the limit of their adaptation capacity [13]. In contrast to fibroblasts and despite a more substantial increase in mtDNA abundance, eIF2B-mutant astrocytes implement a more complex adaptation approach and yet their metabolic stress is not completely relieved, thus leading to functional consequences. This might be due to the heavy physiological burden normal astrocytes experience, given their role as the nervous system homeostasis keepers. Astrocytes are involved in multiple functions which include energy storage, ion and water homeostasis, synthesis and secretion of neuroprotective factors, synapse formation and modulation, uptake and detoxification of glutamate, and tissue repair [38]. 

Glucose is the major source of energy in the brain and astrocytes are the only brain cells that can store glucose as glycogen. In contrast to neurons that rely on a high rate of oxidative mitochondrial metabolism, astrocytes have a unique capacity to adapt to metabolic challenges. Although astrocytes characteristically rely on glycolysis, they possess almost as many mitochondria as neurons [39,40] and perform oxidative metabolism using various substrates as fuels in addition to glucose. To support neuronal activity, astrocytes undergo glycogenolysis when needed, thus allowing neurons to uptake larger quantities of glucose from the blood [41,42,43,44]. Moreover, astrocytes uptake glutamate from nerve synapses and use it for conversion into glutamine, or as a substrate for oxidative ATP production in their mitochondria [45,46,47,48]. The high density of mitochondria in fine astrocytic processes encompassing synapses is thought to provide sufficient ATP via oxidative capacity to account for K^+^ homeostasis and uptake and metabolism of glutamate [39]. In addition to their extensive role in supporting neuronal activity, astrocytes undergo activation in response to physiological cues to support brain development and repair. Activation involves energy-consuming molecular and morphological changes necessary for proliferation, migration, synthesis and secretion of cytokines, chemokines and neurotropic factors [49]. Astrocytes are unique for their role as contributors of lipids and cholesterol to oligodendrocytes for myelination in the CNS [50]. Importantly, sterol biosynthesis requires an oxidative environment for its intermediate reactions [51]. To comply with their functional commitments, astrocytes must respond to energy limitation by shaping their adaptation strategy to allow sufficient ATP production. While increase in OXPHOS capacity is preferred due to its high yield of ATP production, sufficient reducing power must be produced to neutralize the accompanied ROS. 

**Figure 8:**
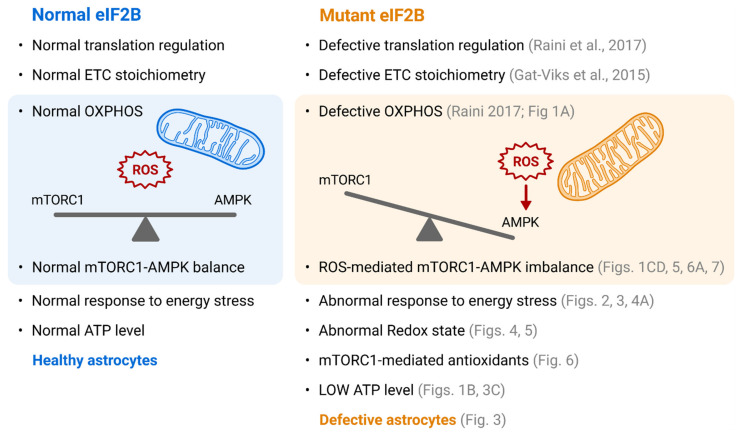
**Blue panel, WT healthy astrocytes.** eIF2B, a master regulator of mRNA translation, is responsible for the correct stoichiometry of electron transfer chain (ETC) complexes, thus allowing effective oxidative phosphorylation (OXPHOS). The cells are able to produce sufficient ATP amounts, as needed. They exhibit normal level of reactive oxygen species (ROS), normal AMPK and mTORC1 activities and are able to normally respond to energy stress. Therefore, astrocytes expressing WT eIF2B are healthy and fully functional. **Orange panel, astrocytes expressing hypo-active eIF2B due to VWMD mutation in Eif2b5 gene.** The cells exhibit defective translation regulation and defective stoichiometry of ETC complexes, resulting in OXPHOS deficit. They suffer from low ability to produce sufficient ATP amounts and compromised ability to adapt to energy stress. They demonstrate ROS-dependent impaired AMPK-TORC1 balance since they require high mTORC1-mediated translation to allow oxidative glycolysis and high level of antioxidants (similarly to cancer cells) which keep their steady-state ROS below normal level. Eif2B5-mutant astrocytes are functionally defective.

In this study, we used glucose starvation to push astrocytes to their metabolic limits in order to expose a ‘hidden phenotype’ caused by mutated eIF2B that affects their adaptation capacity. We discovered that ROS toxicity dictates the level of AMPK-mediated increase in OXPHOS. The NAC-mediated increase in AMPK activity agrees with this notion, since NAC reducing power resolved the barrier to a higher AMPK activity (Figure 5C). The ROS-dependent balance shift towards lower AMPK and higher mTORC1 activities in Mut astrocytes suggests an important role of mTORC1 in redox homeostasis (Figure 1C,D, Figure 2A, Figure 5A,C and Figure 6A,B). The literature reports that cellular redox homeostasis is context-dependent and involves numerous adaptive responses designed to confer a wide spectrum of defensive antioxidant to counteract scenario-dependent redox perturbations [52]. The significant decrease in activity of SIRT1, a mediator of specific antioxidants expression [20] (Figure 2H) supports this view and suggests that astrocytes expressing hypo-active eIF2B entail a different blend of antioxidants supply, which is achieved by mTORC1 activation (Figure 6AB). Since AMPK activity suppresses mTORC1 signaling [53,54], the shift to the lower AMPK activity despite low ATP level in Mut astrocytes (Figure 1A,B, Figure 2A and Figure 3C), agrees with the notion that high AMPK activity is not compatible with the mTORC1-mediated generation of antioxidants. The increased sensitivity of Mut to AICAR although it effectively reduces cellular ROS (Figure 7C,D), supports the conclusion that survival of Mut astrocytes depends on a different route of redox homeostasis. The data implies that an ideal adaptation strategy of Mut astrocytes includes compromised AMPK activity (Figure 1) which supports optimal increase in mitochondrial function, maintenance of active mTORC1/S6K1 axis (Figure 6A) and increase in glycolysis (Figure 3A). The entire take-home message of this study is summarized in Figure 8.

mTORC1 signaling induces the expression of genes encoding enzymes responsible for glycolysis and PPP and co-regulates oxidative PPP with lipid biosynthesis [55,56]. Oxidative PPP supports maintenance of mitochondrial NADPH pool, serving as a powerful reducing power against high ROS levels generated in response to energy stress [57]. While oxidative PPP is one of the most prominent NADPH-producing pathways, lipid synthesis is one of the most NADPH-demanding pathways [58]. It therefore seems that Mut astrocytes share their adaptation strategy with cancer cells, as they utilize aerobic glycolysis to allow diversion of glycolytic intermediates to biosynthetic pathways necessary for cell growth and division, while relying on the mitochondria for energy power [59]. A rise in mTORC1 activity is a common molecular characteristic of the majority of human cancers [60]. Solid tumors acquire radio-resistance via mTORC1-mediated antioxidant activation, which provides them with high-threshold redox homeostasis [31], possibly similar to the redox state of astrocytes expressing hypo-active eIF2B. 

mTORC1 signaling includes facilitation of global 5′cap-dependent translation initiation by direct phosphorylation of eukaryotic initiation factor 4E (eIF4E)-binding proteins (4E-BPs) [36]. A specific sub-class of mRNAs harboring a typical Cytosine Enriched Regulator of Translation (CERT) motif within the 5′UTR, is highly dependent on mTORC1-mediated eIF4E availability. This mRNA subclass is enriched with antioxidants, with FTH1 on the top of the list [35]. The extensive increase in FTH1 expression in Mut astrocytes (Figure 6D) is consistent with activation of mTORC1 signaling to facilitate redox homeostasis. FTH1 is required for combating the toxicity of accumulated ETC-related iron-sulfur clusters during mitochondria biogenesis, since iron catalyzes ROS formation in the presence of oxygen, leading to mitochondrial dysfunction, lipid peroxidation and neurodegeneration [34,61]. Importantly, the second major iron-reducing system is NADPH-dependent [62].

The current study reveals the power of accurate global translation regulation and its robust impact on metabolism and redox homeostasis in astrocytes, providing important insights into the pathophysiology of VWM disease. While the indirect impact of hypoactive eIF2B on the transcriptome, translatome and proteome under normal and stress conditions is inevitable, it should be emphasized that the primary cause is at the mRNA translation level, since eIF2B is a master translation regulator. Given the involvement of astrocytes in a wide range of high-ATP demanding functions, the downstream effects of low ATP levels are expected to be numerous. It seems that although eIF2B-mutant astrocytes use a metabolic program that allows their survival, they pay functional tolls under normal and even more so under further energy stress conditions. Shown here is one example of impaired migration capacity (Figure 3). The increase in mTORC1 activity might also elicit a functional toll since it downregulates autophagy [18]. In support of this possibility is the depressed autophagy of eIF2B-mutant oligodendrocytes [63]. In addition, eIF2B-mutant astrocytes suffer from hypersensitivity to ROS, and demonstrate aberrantly low steady-state ROS level compared to WT astrocytes (Figure 4A,B), which counterintuitively may be viewed as a negative outcome since maintaining certain basal level of ROS in cells is essential for life [64]. Although VWM disease is known to affect mostly white matter, an additional exciting possibility is the probable impact of the impaired oxidative power of eIF2B-mutant astrocytes on their capacity to uptake glutamate and metabolize it. Additional future work, beyond the scope of the current study, is required to clarify all the remaining open mechanistic questions and elucidate the connections to VWMD symptoms. While it is impossible to isolate the primary event from all downstream regulatory layers, it may be possible to identify a specific target for therapeutic intervention. For example, direct targeting of eIF2B by ISRIB, an ISR inhibitor [65], may be useful since eIF2B-mutant cells exhibit hyper-active ISR [66,67], and since ISR has been implicated in regulation of mTOR signaling in a ROS-dependent manner [68]. 

## Data Availability

The data presented in this study are available on request from the corresponding author.

## Supplementary Materials

The following are available online at https://www.mdpi.com/article/10.3390/cells10081858/s1, Figure S1: Purity of primary astrocytes cultures; Figure S2: Confirmation of the abnormal phenotype of Mut astrocytes; Figure S3: PGC1α subcellular localization (supplementary to Figure 2B); Figure S4: GS effect on FTH1 protein level.

## Abbreviations

ACC	acetyl-CoA carboxylase
AMP	activated protein kinase–AMPK
Ars	Arsenite
DMEM-GS	DMEM without glucose
DMEM-HG	DMEM high glucose (25 mM)
ETC	electron transfer chain
eIF2B	eukaryotic translation initiation factor 2 (protein complex)
Eif2b5	symbol of murine gene encoding the catalytic subunit of eIF2B
FAO	fatty acid β-oxidation
FTH1	ferritin heavy chain 1
GS	glucose starvation
mtDNA	mitochondrial DNA
Mut	Eif2b5^R132H/R132H^
NAC	N-acetyl-L-cystein
OXPHOS	oxidative phosphorylation
PGC1α	peroxisome proliferator-activated receptor gamma coactivator 1α
PPP	pentose phosphate pathway
SIRT1	NAD-dependent deacetylase sirtuin-1
VWMD	vanishing white matter disease
WT	wild-type
