# Supplementary material for: The Energy Status of Astrocytes Is the Achilles’ Heel of eIF2B-Leukodystrophy
Source: Cells. 2021 Jul 22;10(8):1858. doi: 10.3390/cells10081858 (PMC8393801; doi:10.3390/cells10081858)

Supplementary Figures

Figure S1

Purity of primary astrocytes cultures

Left panel: representative analysis of a sample of cells following their isolation from the brains of newborn mice. Cells stained with APC-conjugated ACSA1 antibodies. Gray, unstained control. Shown are ACSA1-positive WT (blue) and Mut (orange) primary astrocytes (~94% of the isolated cells in the sample).

Right panel: representative image of the primary astrocytes culture, stained with DAPI (blue) and antibodies specific for GFAP (green). Scale bar, 10 µm.

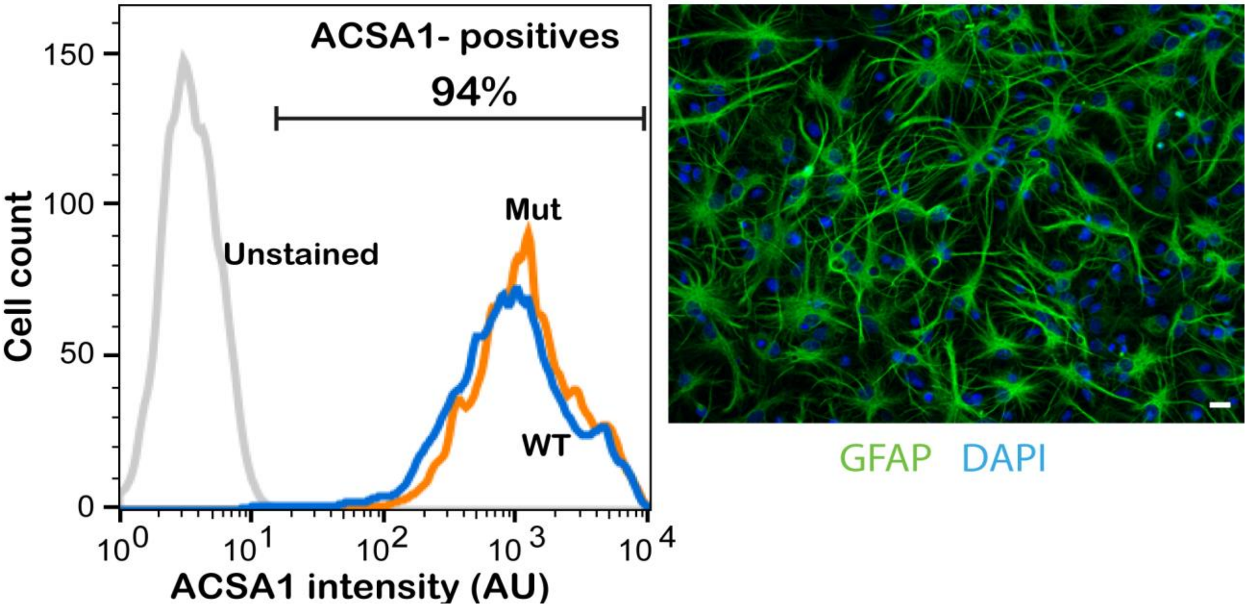

Figure S2

Mut astrocytes exhibit higher MitoTracker Deep Red staining

WT (blue) and Mut (orange) primary astrocytes were incubated for 48 hr in DMEM-HG medium followed by MitoTracker® Deep Red FM staining and flow cytometry analysis. Left panel: shown is a representative experiment. Gray and black lines, unstained controls.

Right panel: Bars represent values of mean MitoTracker intensity ± SEM of 3 independent experiments, relative to WT. *p<0.03; Student's t-test.

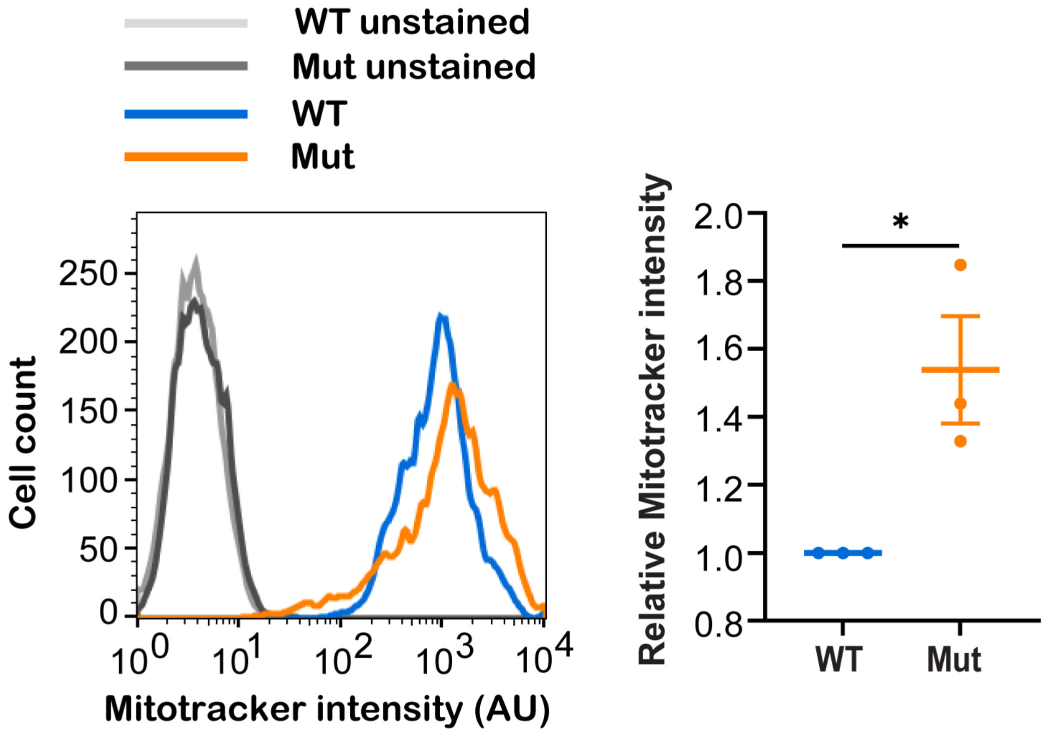

Figure S3

PGC1α subcellular localization (supplementary to Figure 2B)

Shown are representative images of Mut primary astrocytes cultured in DMEM-HG medium (left) or following incubation with DMEM-GS medium for 4 hr (right). The cells were stained with Hoechst (dark blue) and PGC1α antibodies (green). Scale bar, 10 μm. Merged images are shown. Note that glucose starvation increases nuclear localization of PGC1α (as indicated by nuclear light blue color upon decrease of the cytoplasmic PGC1α green signal).

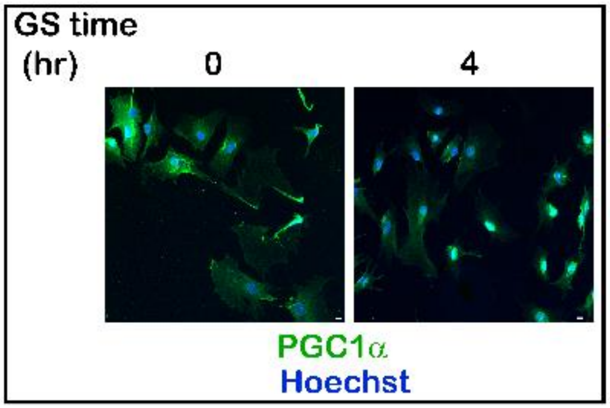

Figure S4

GS effect on FTH1 protein level.

Immunoblot analyses of FTH1 protein level in WT (blue) and Mut (orange) primary astrocytes incubated for 48 hr in DMEM-HG or DMEM-GS medium. A representative blot is shown. Bars represent average ± SEM of FTH1 per actin ratio ± SEM of ≥3 repetitions, normalized to WT-HG. ***$p < 0.001$; ****$p<0.0001$ Student's t-test.

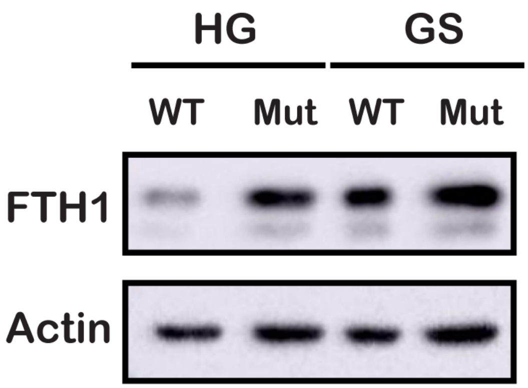

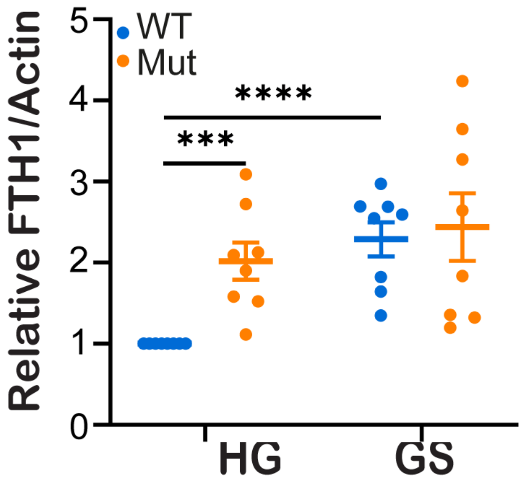

Supplement: Supplementary file 1 [file cells-10-01858-s001.zip › cells-1213278-supplementary.pdf]
